# Supplementary material for: Association of Circulating Neutrophils with Relative Volume of Lipid-Rich Necrotic Core of Coronary Plaques in Stable Patients: A Substudy of SMARTool European Project
Source: Life (Basel). 2023 Feb 2;13(2):428. doi: 10.3390/life13020428 (PMC9958623; doi:10.3390/life13020428)
Supplement: Supplementary file 1 [file life-13-00428-s001.zip › life-2148831-supplementary.pdf]

**Table S1.** *Bivariate linear correlation between the individual RFI values of Neutrophil-Platelet-Aggregates (NPAs, complexes neutrophil-CD41a+) and the corresponding neutrophil receptor expression (RFI), in the selected group of patients (n° = 55).*

| | ***p*-Value** | **R** |
|---|---|---|
| CX3CR1 (RFI) | 0.0163 | 0.323 |
| CD18 (RFI) | < 0.0001 | 0.554 |
| CCR5 (RFI) | < 0.0001 | 0.707 |
| CCR2 (RFI) | 0.0048 | 0.375 |

$p < 0.05$: statistically significant; R = linear regression coefficient. RFI = relative fluorescence intensity. Abbreviations: CX3CR1, C-X3-C motif chemokine receptor type 1 (also fractalkine receptor); CCR5, C-C motif chemokine receptor type 5; CCR2, C-C motif chemokine receptor type 2; CD, cluster of differentiation.
